# WASH practices and its association with nutritional status of adolescent girls in poverty pockets of eastern India

**DOI:** 10.1186/s12905-019-0787-1

**Published:** 2019-07-05

**Authors:** Aparajita Chattopadhyay, Vani Sethi, Varsha P. Nagargoje, Abhishek Saraswat, Nikita Surani, Neeraj Agarwal, Vikas Bhatia, Manisha Ruikar, Sourav Bhattacharjee, Rabi N. Parhi, Shivani Dar, Abner Daniel, H. P. S. Sachdev, C. M. Singh, Rajkumar Gope, Vikash Nath, Neha Sareen, Arjan De Wagt, Sayeed Unisa

**Affiliations:** 1 International Institute for Population Sciences, Mumbai, Maharashtra India; 2 Nutrition Section, UNICEF India Country Office, 73 Lodhi Estate, New Delhi, India; 3 All India Institute of Medical Sciences (AIIMS), Patna, India; 4 All India Institute of Medical Sciences (AIIMS), Bhubaneswar, India; 5 All India Institute of Medical Sciences (AIIMS), Raipur, India; 6 UNICEF India, Field Office Odisha, Bhubaneswar, India; 7 UNICEF India, Field Office Bihar, Patna, India; 8 Sitaram Bhartia Institute of Science and Research, New Delhi, India; 9 Ekjut, Chakradharpur, Jharkhand India; 10 Independent Consultant, New Delhi, India

**Keywords:** Adolescent nutrition, Menstrual hygiene, Open defecation, WASH, Stunting, BMI, MUAC

## Abstract

**Background:**

Water, Sanitation, and Hygiene (WASH) practices may affect the growth and nutritional status among adolescents. Therefore, this paper assesses WASH practices and its association with nutritional status among adolescent girls.

**Methods:**

As a part of an intervention programme, this study is based on baseline cross-sectional data. It was conducted between May 2016–April 2017 in three Indian states (Bihar, Odisha, and Chhattisgarh). From a sample of 6352 adolescent girls, information on WASH practices, accessibility to health services and anthropometric measurements (height, weight and mid upper arm circumference (MUAC)) was collected. Descriptive statistics were used to examine WASH practices, and nutritional status among adolescent girls. Determinants of open defecation and menstrual hygiene were assessed using logistic regression. Association between WASH and nutritional status of adolescent girls was determined using linear regression.

**Results:**

Findings showed 82% of the adolescent girls were practicing open defecation and 76% were not using sanitary napkins. Significant predictors of open defecation and non use of sanitary napkin during menstruation were non Hindu households, households with poorer wealth, non availability of water within household premise, non visit to *Anganwadi* Centre, and non attendance in *Kishori* group meetings. One-third of adolescent girls were stunted, 17% were thin and 20% had MUAC < 19 cm. Poor WASH practices like water facility outside the household premise, unimproved sanitation facility, non use of soap after defecation had significant association with poor nutritional status of adolescent girls.

**Conclusions:**

Concerted convergent actions focusing on the provision of clean water within the household premise, measures to stop open defecation, promotion of hand washing, accessibility of sanitary napkins, poverty alleviation and behavior change are needed. Health, nutrition and livelihood programmes must be interspersed, and adolescents must be encouraged to take part in these programmes.

## Background

India is a country of 243 million adolescents aged 10 to 19 years [1]. Nutritional status of adolescents is an important health issue because the growth during this period is quicker in an individual’s life except infancy. Growths during this phase of life help overall development and provide adequate stores of energy for pregnancy and healthy adulthood. However, the nutrition initiatives in India have been centering on children and women of reproductive age. Few studies provided data on nutritional status of adolescents. In this respect, *SWABHIMAAN* builds the path to comprehend the situation of adolescents in some selected poor districts of three less developed states in India. The nutritional status of adolescent girls is significantly contributing to the nutritional status of the community [2]. In India, 42% of adolescent girls aged 15–19 years have Body Mass Index below 18.5 kg/m^2^ and 50% have anemia. Bihar, Chhattisgarh, and Odisha, these three eastern states of India are known to be poverty-stricken pockets. According to the fourth round of National Family Health Survey (NFHS-4) 2015–2016, 43.2% adolescent girls in Bihar, 38.5% in Chhattisgarh and 37.1% in Odisha have low BMI [3].

Addressing malnutrition among adolescent girls, especially among the early adolescents could be a window of opportunity for ‘catch-up’ growth. This subject is also important for breaking the inter-generational cycle of undernutrition. Undernutrition is directly caused by inadequate dietary intake and/or disease and indirectly related to many factors, including poor water, sanitation and hygiene (WASH) [4, 5]. Thus, reducing the burden of malnutrition among adolescents requires a shift from interventions focusing solely on children and infants to those that reach young girls to improve their nutrition as well as the living environment [6].

Currently ~ 2.3 billion people still lack even a basic sanitation service and nearly 892 million people still practice open defecation worldwide. Around 844 million people around the globe do not have access to improved drinking water sources [7]. In India, as per the NFHS-4 survey (2015–2016), though 10% of the household does not have access to an improved source of drinking water, half (52%) of the household does not use an improved sanitation facility, and 39% of the household practice open defecation [3]. Three states under present study. i.e., Bihar, Chhattisgarh and Odisha are part of government’s Empowered Action Group (EAG) states which lag behingd in demograhic transition and are less developed. In Bihar, Odisha and Chhattisgarh 2, 11, and 9% households do not have access to an improved source of drinking water and 67, 65 and 59% of households practice open defecation [3]. Menstrual cleanliness is another important ‘hygiene’ component. A large number of adolescent girls, especially in rural areas of India and in other countries of Africa and Asia use cloth during menstruation [8–11]. The NFHS-4 (2015–2016) showed that the situation is particularly worse in rural, poverty-stricken and backward regions of the country compared to urban regions [3]. Multidimensional poverty of India, 2018 reveals, almost 46% population are living in severe poverty and are deprived in at least half of the dimensions covered in the index that includes nutrition, health, sanitation, drinking water etc. Poor nutrition is the largest contributor to multidimensional poverty. Pockets of poverty are found across India. But there are limited states (Bihar, Jharkhand, Uttar Pradesh, Madhya Pradesh followed by Odisha and Chhattisgarh) with substantial multidimensional poverty pockets. These states accounted more than half of all poor in India. *Niti Aayog* recently launched the Aspirational Districts programme in (2018) with the aim of fast-tracking the socio-economic status of districts.

A five-year initiative titled *SWABHIMAAN* (meaning: Pride) was launched in 2016, layering essential nutrition interventions on adolescent girls and women through the National Rural Livelihoods Mission (NRLM). Poverty reduction and livelihoods generation initiatives of NRLM provide a suitable platform to layer women’s nutrition interventions. Such initiatives are tapped by *SWABHIMAAN* in three states of eastern India i.e. Bihar, Odisha and Chhattisgarh that stands at the bottommost positions in different development indices. *SWABHIMAAN* covers all the stages of a woman’s life-cycle with heightened nutritional vulnerability that is adolescence, pre-pregnancy (newlyweds), pregnancy and lactation (mothers of children under-two) period.

Several studies [12–15] have addressed the effect of WASH interventions on the incidence of various transmissible diseases and on nutritional status amongst children. **The** ***SWABHIMAAN*** **intervention aims at improving nutrition of adolescent girls through f**ormation of adolescent girls’ clubs/activation of *Sabla* clubs, organizing weekly/fortnightly *Kishori Sakhi* meetings over weekend, providing loans for secondary education of adolescent girls through VO, mobilizing girls for AHD/*Kishori Divas* services and making efforts to reduce child marriage. The current study is based on the base line data of the intervention project to understand the levels and pure effect of WASH and programme components on nutrition. A Cochrane review of WASH interventions on the nutritional status of children reported small benefits of WASH interventions on growth in children under five years of age [5]. However, studies investigating the impact of WASH practices on measures of physical growth and nutritional sufficiency among adolescents in the Indian context are limited. Therefore, the present study assesses the levels of WASH practices and selected nutritional indices of adolescent girls in three tribal pockets of Eastern India. Further it determines the association of WASH and some programme factors on nutritional status of adolescent girls, to guide future interventions.

## Methods

The study is a part of the *SWABHIMAAN* programme which aimed at improving the nutritional status of adolescent girls, pregnant women, and mothers of children less than two years age in three poverty pockets of India (Bihar, Odisha, and Chhattisgarh) dominated by tribal population. As a part of this programme, primary data amongst adolescent girls (10–19 years) were collected between May 2016 and April 2017 from Purnea district (Jalalgarh and Kasba blocks) of Bihar, Angul district (Pallahara block) and Koraput district (Koraput block) of Odisha and Bastar district (Bakawand and Bastar blocks) of Chhattisgarh. Based on the outcome indicators and the change envisaged, a representative sample of 6352 (Bihar: 1704; Odisha: 1727 and Chhattisgarh: 2921) adolescent girls was drawn using the simple random sampling. Fourty 6 % sample population belongs to scheduled tribe. Data were collected by trained teams, consisting of trained supervisors and field investigators.

A pre-tested, structured, bilingual questionnaire in Bihar and Chhattisgarh (English and Hindi), and Odisha (English and Odia) were used to elicit information on: i) socio-demographic profile, ii) WASH practices (main source of drinking water, accessibility of water facility, type of sanitation facility used, practice of open defecation, use of soap after defecation and use of napkins during mensuration), iii) adolescents’ access to health services (accessed health service in last six months, visited *Anganwadi* (rural child care centre in India), accessed any health services, counselling by a frontline health worker, attended any *Kishori* (Adolescent girl) group meetings and able to make decision about own healthcare, iv) anthropometric measurements (weight, height and mid upper arm circumference (MUAC)) were collected using the standard technique [16].

Weight was measured in kilogram (without shoes) using a SECA electronic weighing scale recorded to the nearest 0.1 kg. Height was taken barefooted using stadiometer nearest to 0.1 cm. Mid Upper Arm Circumference was measured in centimeters with a non-stretchable measuring tape nearest to 0.1 cm. The tape was placed firmly but gently on the arm to avoid compression of soft tissue. Quality control checks were conducted for 10% of the interviewed population. The weighing scales and stadiometer were calibrated on a weekly basis prior to data collection with standard weights (1, 2 and 5 kg) and a meter rod (100 cm). The mean standard errors of measurement for height, weight, and MUAC across all the data collection teams were insignificant and ranged between 0.001–0.025 (*p* < 0.10, CI = − 0.004–0.042).

### Variables

Detailed information on dependent and independent variables used in the study is given in this section. Stunting represents the chronic undernutrition that reflects failure to receive adequate nutrition over a long period. Stunting was defined as the height-for-age (HAZ) z-score < − 2 SD and severe stunting as <−3SD. Thinness was defined as BMI < -2SD and severe thinness as <−3SD. Body Mass Index is a measure of weight relative to height, and was calculated (weight in kg/height in m^2^) using WHO growth charts (z-scores) and graded according to WHO classification [17]. The MUAC is the circumference of the left upper arm, measured at the mid-point between the tip of the shoulder and the tip of the elbow and is used for the assessment of nutritional status. Using the data from the *SWABHIMAAN* baseline survey we estimated MUAC cut-offs for adolescent girls (10–19 years) by calculating Youden’s Index. The MUAC value corresponding to the highest value of Youden’s Index was chosen as the cut-off. The MUAC cut-offs for adolescents girls (10–19 years) at <−2SD (thinness) and < −3SD (severe thinness) were < 19 cm and < 17 cm respectively.

WHO norms were used to differentiate between improved and unimproved WASH facilities. Improved sanitation facility includes: flush/pour flush, toilet/ latrine and pit latrines (piped sewer system, septic tank, pit latrine, ventilated improved pit/ biogas latrine, pit latrine with slab, twin pit/ composting toilet). The unimproved sanitation facilities include: flush to somewhere else, pit latrine without slab/ open pit, dry/ service latrine and no sanitation facility/ uses open space or field/ jungle. The households with no sanitation facility were considered practicing open defecation as concept of public toilet facility does not exist in rural India. Improved source of drinking water includes: piped water into dwelling/yard/plot, public tap/standpipe, tube well or borehole, protected dug well, protected spring/ rainwater and community reverse osmosis plant. Additionally, whether the water facility was within (also includes water piped into a dwelling/plot/yard) or outside the premise of the household was also included in the analysis. The menstrual practice was considered hygienic if the girl was using locally prepared napkins or sanitary napkins.

The other covariates included in the study were age (10–14 and 15–19 years); religion was divided into two categories, i.e. Hindu and non-Hindu; caste was categorized into Scheduled Caste (SC), Scheduled Tribe (ST), Other Backward Classes (OBC) and others. Five categories constituted the wealth quintile based on series of assets i.e. poorest, poor, middle, rich and richest. Covariates like currently attending school, engaged in work outside home and earnings in cash were also included in the dichotomous form (Yes/No).

The variable ‘accessing adolescent health services in past 6 months’ denoted girls’ participation in Adolescent Health Day. Adolescent Health Day (AHD) is part of a national strategy, *Rashtriya Kishor Swasthya Karyakaram* (RKSK). The AHD intends to strengthen the adolescent health program and improve preventive services and increase the awareness among adolescents. The variable ‘receiving health service/counseling from frontline health worker’ indicates receipt of any health and nutrition related advice or counseling from AWW, ANM or ASHA. *Anganwadi* Centre is a part of ICDS programme that provides basic nutrition and health facilities. The variable ‘visiting *Anganwadi* Centre’ includes availing facilities like dry ration or take home ration, health check-up, counseling, sanitary napkins, or medicines. *Kishori* group is the adolescent girls’ club organized by *Kishori Sakhi* addressing issues specific to adolescent girls using participatory learning and action cycle methodology.

### Statistical analysis

Descriptive statistics were used to examine the characteristics of adolescent girls, their WASH practices, nutritional status, and participation in health and nutrition programmes. The multivariate logistic regression with 95% confidence interval (CI) was used to determine the predictors of open defecation and menstrual hygiene practices of adolescent girls. In two logistics regressions, considering open defecation and menstrual hygiene as dependent variable, independent variables were socio-demographic factors, WASH facilities, participation in health interventions and programmes. Linear regression was employed to assess the relationship between the nutritional status of adolescent girls and WASH practices. Here, stunting, BMI and MUAC were considered as dependents variables, and WASH facilities and practices, participation in health and nutrition programmes were considered as independent variables. All the analyses were performed using SPSS version 20.

## Results

### Characteristics of adolescent girls

A total of 6352 adolescent girls (Bihar: 1704, Odisha: 1727 and Chhattisgarh: 2921) were included in the study. The socio-demographic characteristics of the adolescent girls were reflective of underprivileged populations. Majority of the adolescent girls were Hindus (82%) and belonged to the scheduled tribe (54–65% in Odisha and Chhattisgarh) and other backward classes (68% in Bihar). Nearly one-third of girls were not attending school and were engaged in work outside the home (Table 1).

**Table 1:** Percentage of adolescent girls by socio-demographic characteristics, WASH practices, and participation in programmes

Characteristics	Bihar (*N* = 1704)	Odisha (*N* = 1727)	Chhattisgarh (*N* = 2921)	Total (*N* = 6352)
Age				
10–14 years	63.2	51.2	55.5	56.4
15–19 years	36.8	48.8	44.5	43.6
Religion				
Hindu	41.9	95.3	98.3	82.3
Non-Hindu	58.1	4.7	1.7	17.7
Caste				
Scheduled Caste (SC)	19.1	15.4	2.4	10.4
Scheduled Tribe (ST)	4.9	54.1	65.1	46.0
Other Backward Classes (OBCs)	68.3	22.6	27.9	37.3
Others	7.7	7.9	4.6	6.3
Wealth quintile				
Poorest	6.2	32.2	21.2	20.0
Poor	13.2	23.3	21.7	20.0
Middle	24.5	16.4	19.5	20.0
Rich	31.8	15.0	16.1	20.0
Richest	24.4	13.2	21.5	20.0
Currently attending school				
Yes	80.6	57.3	74.8	71.6
No	19.4	42.7	25.2	28.4
Engaged in work outside home				
Yes	11.7	21.4	30.4	23.0
No	88.3	78.6	69.6	77.0
Earnings in cash^a^				
Yes	88.9	98.3	98.9	97.4
No	11.1	1.7	1.1	2.6
Main source of drinking water				
Improved^b^	99.9	83.0	95.0	93.1
Unimproved	0.1	17.0	5.0	6.9
Accessibility to water facility				
Within premises^c^	0.1	1.7	7.3	3.8
Out of premises	99.9	98.3	92.7	96.2
Type of sanitation facility used				
Improved^d^	18.1	12.7	16.2	15.7
Unimproved^e^	81.9	87.3	83.8	84.3
Practice open defecation^f^				
Yes	79.3	83.0	83.2	82.1
No	20.7	17.0	16.8	17.9
Uses soap after defecation				
Yes	72.8	63.1	74.8	71.1
No	27.2	36.9	25.2	28.9
Uses sanitary napkin^g^ during menstruation^h^				
Yes	11.2	37.4	24.8	23.3
No	88.8	62.6	75.2	76.7
Accessed adolescent health services in last six months				
Yes	2.5	7.9	13.8	9.2
No	97.5	92.1	86.2	90.8
Visited *Anganwadi*Centre (AWC) for service				
Yes	3.4	27.0	37.1	25.3
No	96.6	73.0	62.9	74.7
Accessed any health service/counselling from frontline health worker				
Yes	2.7	20.1	18.0	14.5
No	97.3	79.9	82.0	85.5
Attended any *Kishori* group meetings				
Yes	2.1	9.0	5.9	5.7
No	97.9	91.0	94.1	94.3
Able to make decision aboutown healthcare				
Yes	25.6	62.3	36.8	40.7
No	74.4	37.7	63.2	59.3

Note:

^a^Includes working women (1449) only. Hence sample does not match with total sample (6352)

^b^Improved source of drinking water as per WHO norm includes piped water into dwelling/yard/plot, public tap/standpipe, tube well or borehole, protected dug well, protected spring, rainwater, and community RO plant

^c^Water facility within premises includes water piped into a dwelling, plot or yard

^d^Improved sanitation facility as per WHO norms include flush or pour flush toilet/latrine to: piped sewer system, septic tank, pit latrine, ventilated improved pit (VIP)/biogas latrine, pit latrine with slab, twin pit/composting toilet

^e^Unimproved sanitation facilities includes: flush to somewhere else, pit latrine without slab/open pit, dry/service latrine

^f^Open defecation represents household that have no sanitation facility and defecate in open spaces or field/jungle

^g^Sanitary napkin refers to a sanitary pad or any locally prepared sanitary napkin

^h^Includes menstruating girls (4869) only hence sample does not match total sample (6352)

### WASH practices by adolescent girls and receipt of services

Overall 93% of the adolescent girls used an improved source of drinking water. However, a large proportion of girls (96%) had the water facility outside the household premises. Eighty two percent of them had no sanitation facility in the household, 29 % were not using soap for washing hands after defecation; this proportion was highest in Odisha (37%) as compared to the other two states. Majority of the girls (77%) were not using sanitary napkins during menstruation (Table 1).

**Fig. 1:**
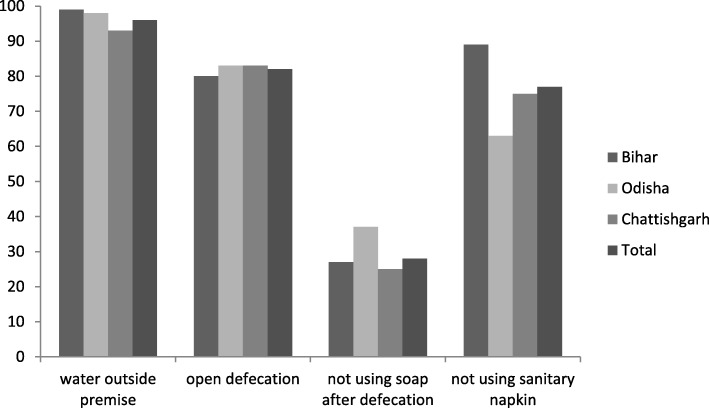
Selected WASH practices in the study area

Figure 1 shows the access to water outside household premise practice of open defecation and selected unhygienic practices in the study area. Almost all the households in Bihar had water facility outside the household. Around 83% households in Chhattisgarh practiced open defecation which is highest among the states under study. Thirty-seven percent households in Odisha did not use soap after defecation and nearly 89% girls did not use sanitary napkin during menstruation.

Only 9% of the girls accessed adolescent health services in the last six months preceding the survey and 25% visited *Anganwadi* Centre (AWC) for availing any services. Majority of the girls (85%) had not accessed any service/ counseling from a frontline health worker. Most of them (94%) reported that they had not attended *Kishori* group meetings in the past six months. Almost half of the girls reported that they could not make decisions regarding their healthcare (Table 1).

### Determinants of open defecation and use of sanitary napkins

Adolescents from non-Hindu religion (OR: 2.36, 95% CI 1.87–2.97), who belonged to scheduled caste (OR: 1.34, CI 0.99–1.83) or scheduled tribe (OR: 1.60, CI 1.23–2.08), poorer in wealth status (OR: 2.35–4.06, CI 1.95–5.08), not having access to water within the household premise (OR: 1.73, CI 1.28–2.35), and not visiting AWC for any services (OR: 1.48, CI 1.24–1.75) were more likely to defecate in open (Table 2). For menstrual hygiene, girls belonging to Hindu religion (OR 3.34, CI 2.37–4.71), with better wealth quintile (OR: 1.31–3.17, CI 0.96–4.30), attending schools (OR 2.19, CI 1.80–2.66), engaged in paid work outside home (OR 0.72, CI 0.58–0.88), able to make decisions about their healthcare (OR: 1.21, CI 1.02–1.45), using improved sanitation facilities (OR: 1.34, CI 1.07–1.68), and who attended *Kishori* group meetings (OR 1.57, CI 1.10–2.26) were more likely to use sanitary napkins during menstruation (Table 3).

**Table 2:** Logistic regression showing determinants of open defecation

Characteristics	Odds ratio	CI (95%)
Socio-demographic		
Religion		
Hindu®		
Non-Hindu	2.36***	1.87–2.97
Caste		
Others®	1.00	
Other Backward Classes (OBCs)	1.20	0.93–1.55
Scheduled Caste (SC)	1.34*	0.99–1.83
Scheduled Tribe (ST)	1.60***	1.23–2.08
Wealth quintile		
Richest®		
Rich	2.35***	1.95–2.84
Middle	2.76***	2.26–3.37
Poor	3.49***	2.81–4.32
Poorest	4.06***	3.25–5.08
Currently attending school		
Yes®		
No	1.13	0.95–1.35
Engaged in paid work outside home		
Yes®		
No	0.85	0.70–1.04
Able to make decision aboutown healthcare		
Yes®		
No	0.96	0.82–1.12
WASH Practices and Programme Participation		
Access to water facility		
Within premises®		
Out of premises	1.73***	1.28–2.35
Accessed adolescent health services organized by health department		
Yes®		
No	1.03	0.79–1.34
Visited *Anganwadi*Centre (AWC) for any service		
Yes®		
No	1.48***	1.24–1.75
Accessed any health service or counselling from a frontline health worker		
Yes®		
No	0.97	0.78–1.21
Attended any *Kishori* group meetings		
Yes®		
No	1.04	0.76–1.42

Note:® - Reference category; *CI* confidence interval, ***p < 0.01, *p < 0.10

**Table 3:** Logistic regression showing predictors of sanitary napkin use

Characteristics	Odds ratio	CI (95%)
Socio-demographic		
Religion		
Non-Hindu®		
Hindu	3.34***	2.37–4.71
Caste		
Scheduled Tribe (ST)®		
Scheduled Caste (SC)	0.87	0.63–1.19
Other Backward Classes (OBCs)	0.85	0.68–1.05
General	0.95	0.65–1.37
Wealth quintile		
Poorest®		
Poor	1.31*	0.96–1.79
Middle	1.52***	1.13–2.05
Rich	2.25***	1.67–3.03
Richest	3.17***	2.33–4.30
Currently attending school		
No®		
Yes	2.19***	1.80–2.66
Engaged in paid work outside home		
No®		
Yes	0.72***	0.58–0.88
Able to make decision about own healthcare		
No®		
Yes	1.21**	1.02–1.45
WASH Practices and Programme Participation		
Type of sanitation facility		
Unimproved/Open Defecation^a^®		
Improved	1.34**	1.07–1.68
Accessed adolescent health services organized by health department		
No®		
Yes	0.91	0.67–1.25
Visited *Anganwadi* Centre (AWC) for any service		
No®		
Yes	1.07	0.87–1.32
Accessed any health service or counselling from a frontline health worker		
No®		
Yes	0.84	0.65–1.08
Attended any *Kishori* group meetings		
No®		
Yes	1.57**	1.10–2.26

Note:® - Reference category; *CI* confidence interval, ***p < 0.01, **p < 0.05, *p < 0.10

^a^In this category majority of the households practiced open defecation (97.8%) whereas only 2.2% used unimproved sanitation facilities

### Nutritional status of adolescent girls

**Fig. 2:**
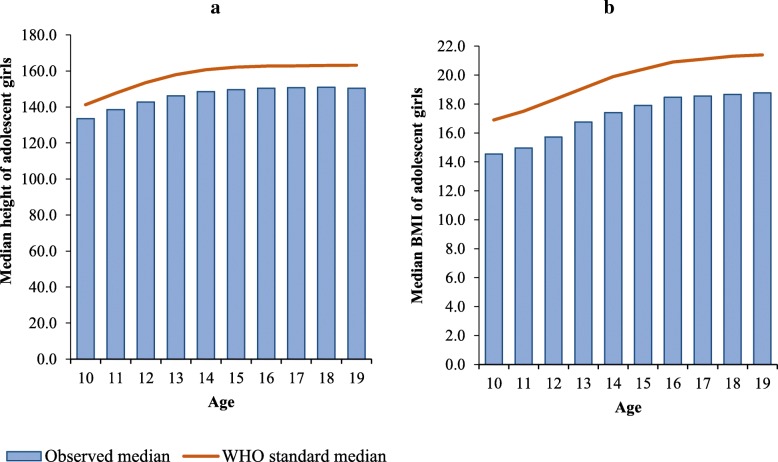
WHO standard median and observed medians for (**a**) height and (**b**) BMI among adolescent girls

About one-third of the girls were stunted, 17% had BMI z-score < −2SD (thin), and 20% had MUAC below 19 cm (Table 4). Overall, the prevalence of stunting was higher among late adolescent girls aged 15–19 years. However, low BMI (< 18.5 kg/m^2^) and MUAC were more prevalent among young adolescent girls aged 10–14 years. As compared to Chhattisgarh and Odisha, nutritional status of adolescent girls in Bihar was worse; 43% girls were stunted, 27% were thin, and 33% had low MUAC. A difference was found in the observed median height of girls and the WHO standard for median height for each age group (Fig. 2a). The gap in standard and observed median height increased with age. Similarly, there was a considerable gap in the WHO standard median BMI and observed median BMI of girls in each age group (Fig. 2b).

**Table 4:** Percentage of early (10–14 years) and late (15–19 years) adolescent girls by stunting, BMI, and MUAC

	Bihar			Odisha			Chhattisgarh			Total		
	10–14	15–19	N	10–14	15–19	N	10–14	15–19	N	10–14	15–19	N
Stunting												
< −2SD	44.1	40.3	42.7	23.9	46.6	35.0	21.9	34.9	27.7	28.9	39.7	33.6
<−3SD	17.5	15.1	16.6	4.6	9.5	7.0	3.5	4.7	4.0	7.9	8.4	8.1
N	1032	596	1628	879	839	1718	1615	1300	2915	3526	2735	6261
BMI												
< −2SD	27.2	26.1	26.8	16.5	8.2	12.5	17.4	10.5	14.3	20.0	13.2	17.1
<−3SD	7.7	6.6	7.3	3.8	1.5	2.7	3.6	1.8	2.8	4.8	2.7	3.9
N	1030	593	1623	879	839	1718	1613	1297	2910	3522	2729	6251
MUAC												
< 19 cm	47.6	7.0	32.7	29.7	1.2	15.8	27.5	1.7	16.0	34.1	2.7	20.4
< 17 cm	20.7	2.1	13.9	7.5	0.1	3.9	5.3	0.2	3.1	10.5	0.6	6.2
N	1075	625	1700	879	840	1719	1615	1300	2915	3569	2765	6334

### Relationship between WASH and nutritional status

Water facility outside the household premise had a significant association with stunting and low BMI. Girls who accessed water from outside the household were more likely to be stunted (*p* < 0.01) and were thin (*p* < 0.10), particularly the young adolescent girls (*p* < 0.05). Open defecation had a significant stunting (*p* < 0.05) and low MUAC (< 19 cm) (*p* < 0.01). The girls, not using soap after defecation, were more likely to have low MUAC, especially young adolescent girls (*p* < 0.01). Not attending health services organized by the health department had a considerable negative association with low MUAC (*p* < 0.10). Similarly, non visit to the AWC had a negative impact on BMI and MUAC of older adolescent (*p* < 0.05). Not attending *Kishori* group meetings was also negatively associated with BMI (*p* < 0.05) and low MUAC (*p* < 0.01) among young adolescent girls aged 10–14 years (Table 5).

**Table 5:** Linear regression showing determining the nutritional status of adolescent girls

Variables	Stunting			BMI			MUAC		
	Coefficients								
	10–14 yrs	15–19 yrs	Total	10–14 yrs	15–19 yrs	Total	10–14 yrs	15–19 yrs	Total
Accessibility to water facility									
Within premises®	1.00	1.00	1.00	1.00	1.00	1.00	1.00	1.00	1.00
Out of premises	−0.20**	−0.16*	−0.19***	−0.20**	−0.03	−0.14*	−0.29	−0.06	−0.27
Households’ sanitation facility									
Improved®	1.00	1.00	1.00	1.00	1.00	1.00	1.00	1.00	1.00
Unimproved/Open Defecation	−0.10*	− 0.04	− 0.08**	0.01	0.02	0.02	−0.32***	− 0.09	− 0.28***
Uses soap after defecation									
Yes®	1.00	1.00	1.00	1.00	1.00	1.00	1.00	1.00	1.00
No	−0.05	−0.01	− 0.01	− 0.05	0.00	− 0.03	− 0.26***	−0.17*	− 0.31***
Uses sanitary napkin during menstruation									
Yes®	NA	1.00	NA	NA	1.00	NA	NA	1.00	NA
No	NA	−0.04	NA	NA	−0.05	NA	NA	−0.04	NA
Accessed adolescent health services organized by health department									
Yes®	1.00	1.00	1.00	1.00	1.00	1.00	1.00	1.00	1.00
No	0.08	0.05	0.08	−0.03	0.08	0.01	−0.29	−0.03	− 0.26*
Visited *Anganwadi*Centre (AWC) for any service									
Yes®	1.00	1.00	1.00	1.00	1.00	1.00	1.00	1.00	1.00
No	−0.08	−0.03	− 0.01	−0.08	− 0.09**	−0.08**	− 0.52***	−0.20**	− 0.66***
Accessed any health service/counselling from a frontline health worker									
Yes®	1.00	1.00	1.00	1.00	1.00	1.00	1.00	1.00	1.00
No	0.06	0.06	0.06	0.09	0.05	0.08	0.33	0.18	0.30
Attended any *Kishori* group meetings									
Yes®	1.00	1.00	1.00	1.00	1.00	1.00	1.00	1.00	1.00
No	0.02	−0.04	0.01	−0.22**	−0.00	−0.09	−0.62***	−0.07	− 0.40**

Note:® - Reference category; ***p < 0.01, **p < 0.05, *p < 0.1

## Discussion

The scourge of under-development is confined to certain pockets in India, especially in the central and eastern states where our study area is located. This is seen as an opportunity for backward districts that lack basic amenities, infrastructure, health facilities, etc., to grow. Lack of sanitation, in particular, is strongly correlated with acute malnutrition and stunting (low height for age) [18]. Thus scientific evidence is the need of the hour to strengthen programme implementation. The association between WASH and morbidities has been extensively scrutinized; however, the literature on the inter-relationship between WASH and malnutrition provide mixed results. In this context, the paper is a valuable input that explores the nutritional status of the adolescent girls in selected poverty pockets of rural India. It explores basic facilities and practices like drinking water, sanitation facility, hand washing and use of sanitary napkins. Further, it shows the association of WASH practices and programme impacts on adolescent nutrition. This paper examined the inter-relationship between WASH and nutritional status of adolescent girls.

### Four important inferences for programme acceleration can be drawn from the results

*Firstly*, easy access to water as established in the study is an important determinant to reduce malnutrition amongst adolescents. In the present study, overall 93% of the adolescent girls used an improved source of water. Similarly, NFHS-4 survey (2015–2016) also reported that 90% of the household have access to an improved source of drinking water. However, 96% water facility is available outside household premise. Literature already proved that treatment and safe storage of drinking water in the household reduce the risk of diarrheal disease by 30–40% [18]. It could be suggested that improved nutritional status amongst adolescent girls could be achieved through continued efforts to deliver an efficient supply of water within the household premise [3].

*Secondly*, the findings of the study must be linked to interventions such as the *Swachh Bharat* (Clean India) Mission to implement evidence-based programme. The focus should also be on strengthening national institutions, fostering strong private sector participation, and enabling behaviour change, rather than merely building toilets. Improved sanitation and personal hygienic practices are crucial for reducing the risks of communicable diseases and improving public health [19]. Open defecation poses a serious threat to the health of children/adolescents in India and especially in the rural areas and poor households. India remains one of the countries with highest rates of open defecation (prominent especially in rural areas), despite the recent growth and poverty reduction; the percentage of such practice is still high compared to rest of the South Asia and the world [7].

Overall in the present study, 82% (Bihar: 79%, Odisha: 83% and Chhattisgarh: 83%) of the adolescent girls were practicing open defecation. NFHS-4 survey (2015–2016) reported that 39% of the household practice open defecation with 67, 65 and 59% in states of Bihar, Odisha, and Chhattisgarh [3]. Similarly, the National Sample Survey also reported that more than half of India’s rural population (52%) defecates in the open [20]. Diarrheal related deaths amongst adolescents are reported to be amongst the top ten for the age group of 10–19 year and second among the age group of 10–14 year globally [19]. Several studies have documented that open defecation is a significant constraint on growth in South Asia [21–25]. Provision of sanitary facility and access to water are fundamental rights that need focused attention.

*Thirdly*, a policy level concrete decision to universalize the Menstrual Hygiene Scheme (MHS) is much needed. About three fourth of the adolescent girls in our study are not using sanitary napkins. Menstrual Hygiene Management (MHM) is a problem for adolescent girls in low and middle-income countries (LMICs), particularly when attending school [26]. Menstrual cleanliness is a crucial ‘hygiene’ component. A large number of adolescent girls, especially in rural areas, use cloth during menstruation [10, 11, 27] like many other countries of Africa, South Asia, and West Asia [8, 9, 28]. Cloths are traditionally used to absorb menstrual flow; they are cheaper and create less environmental pollution, but are gradually being replaced by disposable sanitary napkins, particularly in urban areas. Cleaning and drying cloth is a problem if girls lack water, privacy and a drying place [29, 30]. Such unhygienic practices hinder the proper nutrition and health of girls and are needed to be changed [31]. As revealed in our study, girls who used sanitary napkins were more likely to belong to rich wealth quintile, attended school, and were exposed to *Kishori* group meetings. In India, as per the MHS implemented in some states, sanitary napkins are made available to rural adolescent girls through the ASHA worker [32]. Hence, the issue of cost and access to sanitary napkins seems to be unjustifiable; however, further research is needed on quality and distribution of napkins supplied by ASHA. Concentrated efforts are required to improve information, education, and communication (IEC) activities around the same at available platforms, like schools, AWCs, or through ASHA. During these IEC sessions, adolescent girls also need to be informed about the safe and hygienic sanitary practices and associated health outcomes. Apart from education, awareness, resources and the freedom to choose what material the girls want to use, availability of sanitation facilities, and water availability within premise are a critical area to consider while evaluating sanitary napkin use.

*Fourth*, our study established that WASH practices have a significant association with the nutritional status of adolescent girls (girls who accessed water from outside, used unimproved sanitation facility and were not using soap after defecation were more likely to be stunted, thin and had low MUAC (< 19 cm)). Supporting these findings, many studies have shown that access to water [33, 34] and proper sanitation and hygiene [25, 35] have a significant association with nutritional status of adolescent girls, even after controlling all other vital factors like disease and food intake. Thus, programmes for WASH need to be strengthened, especially in low resource settings. Convergence between health, nutrition, and WASH is requiredto address the problem of malnutrition comprehensively. During community health campaigns and door to door health visits, promoting both improved nutrition and WASH practices will save resources. Positive nutritional outcomes are dependent on WASH interventions and nutrition actions. In the present study, about one-third of adolescent girls were stunted, 17% were thin and 20% had MUAC < 19 cm. Several studies have reported a high prevalence of stunting and thinness amongst adolescent girls [23–25]. Observational studies have found associations between the frequency of open defecation and prevalence of stunting [4]. An analysis of several demographic and health surveys (DHS) in 65 countries reported that open defecation explains over half of the variation in average child height between countries [36]. Another analysis of 171 surveys in 70 LMICs found that increasing access to and use of improved sanitation and improved water sources reduced the risk of stunting [37]. Poor WASH conditions leads to additional burden of undernutrition. The present study found that poor WASH practices (water facility outside the premise, unimproved sanitation facility, not using soap after defecation) and non-participation in health and nutrition services had a significant association with nutritional status of adolescent girls. Similarly, studies reported that lack of drinking water availability in household, poor sanitation and personal hygiene, was significantly associated with high morbidities and poor nutritional status of adolescent [23, 25, 38, 39]. Overall poor WASH practices are identified as a salient factor in determining the overall nutritional status of adolescents [21, 40, 41].

The strength of the present study is that it is a community based survey, conducted on a plausibly large sample size with good quality control and monitoring. The sample was systematically drawn from deprived rural areas which are hotspots for programmes and interventions aimed at improving nutrition. Also, studies on WASH practices and nutritional status of adolescent girls’ aged 10–19 years are rare in India. However, there are also a few caveats, because of the cross-sectional nature of the study, conclusions related to cause and effect cannot be drawn. Also, sample being selected only from three geographical areas is another limiting factor, restricting the generalisability of the results.

### Limitations

We acknowledge that the study is in the half way mark from its larger project objectives. It is based on the baseline survey of an intervention study that is yet to estimate the effects of its intervention. Intermittent data is not available to measure the impact of series of programmes that the project has aimed for. Further, in our analysis, we did not include all known factors that explain health of an adolescent like food intake, disease incidence, health facility availability and utilization, functioning of public distribution etc. due to data limitation. The aim here is to understand pure effect of WASH and selected programme on adolescent health from programmatic point of view. In a large scale survey, it is also difficult to quantify social taboos, quality of toilets, cultural practices, quality and quantity of food availability through public distribution, intra household resource distribution, economic and social inequality, etc. that also determine nutritional health of a young girl. Further, as all the adolescent girls were unmarried, the survey questions did not emphasize the reproductive health aspects of these girls, considering the socio-cultural taboos in rural India.

## Conclusions

WASH practices have a significant association with the nutritional status of adolescent girls. Therefore, there is a need to spread awareness among people in low resource settings regarding the assocation of poor WASH practices and poor nutritional status. From a programmatic point of view, policies related to economic empowerment and health must be strengthened, encouraging visits to the AWC through *Anganwadi* worker/ ASHAs, encouraging adolescent girls to attend *Kishori Diwas* by creating awareness in the community, and emphasizing focused efforts on improving education through strategies such as *Beti Bachao, Beti Padhao* (Save girl child, educate a girl child). Convergence between health, nutrition, and WASH can expansively and inclusively address malnutrition. National Rural Livelihoods Mission (NRLM) also needs to extend services to adolescent girls who are the future mothers.

Moreover, programmes for WASH need to be strengthened, especially in low resource settings. Realization of rights of poor and marginalized people for sufficient clean water also requires utmost attention. Through the National Rural Health Mission (NRHM), the government of India has been trying to reach out to women/girls with low cost branded sanitary napkins. Ministry of Women and Child Development (MoWCD) is also running SABLA programme to improve their nutrition, health status and health knowledge that includes awareness about health, hygiene, nutrition. Demand generation through community mobilization and system strengthening for efficient supply of water, sanitation, hygienic products, and awareness generation are to be realized as a sustainable solution to reduce the burden of undernutrition among adolescent girls.

## Data Availability

The datasets used and/or analysed during the current study are available from the corresponding author on reasonable request.

## Abbreviations

AHD: Adolescent Health Day
AIIMS: All India Institute of medical sciences
AWC: Anganwadi centre
BMI: Body mass index
CI: Confidence interval
CTRI: Clinical trial registry of India
DHS: Demographic and health surveys
HAZ: Height for age
ICMR: Indian council of medical research
IEC: Information education and communication
LMIC: Low middle income countries
MHM: Menstrual hygiene management
MHS: Menstrual hygiene scheme
MoWCD: Ministry of women and child development
MUAC: Mid upper arm circumference
NFHS: National family health survey
NRHM: National rural health mission
NRLM: National rural livelihoods mission
OR: Odds ratio
VO: Village Organisations
WASH`: Water sanitation and hygiene
WHO: `World Health Organization
